# When ‘the show’ cannot go on: An investigation into sports mega-events and responses during the pandemic crisis

**DOI:** 10.1177/10126902211020169

**Published:** 2022-06

**Authors:** Jan Andre Lee Ludvigsen

**Affiliations:** 151597 Liverpool John Moores University, UK

**Keywords:** sports mega-events, coronavirus disease-2019, governance, mega-events, risk

## Abstract

This article examines the relationship between sports mega-events and the coronavirus disease-2019 pandemic. Focusing primarily on the 2020 Summer Olympics and Union of European Football Associations Euro 2020 in football – representing two mega-events that were postponed due to the pandemic – this article explores the emerging discourses from sport governing bodies, and how these organisations communicated their initial responses to the pandemic between February and May 2020. The article takes a digital qualitative research approach and draws upon frame analysed media sources and public communications. As it proceeds, this article first illuminates how global sports entered a temporary standstill and, second, how sport governing bodies positioned themselves with regard to responding to the global crisis from within the sporting sphere. Subsequently, this article emphasises how the relevant responses, as communicated by sport governing bodies, reflected the broader reactive and adaptive pandemic responses apparent within socio-political fields.

## Introduction

This article examines the intersections between coronavirus disease-2019 (COVID-19) and sports mega-events (SMEs). Sociologists are encouraged to analyse the global health crisis caused by COVID-19 (Matthewman and Huppatz, 2020), and arguably SMEs – like sports more widely (see Rowe, 2020) – serve as relevant sites for such sociological analysis. That is because SMEs have well-established capacities to reflect or reveal broader social and cultural processes and changes (Boykoff, 2020; Millward, 2017; Roche, 2000). Defined here as ‘large-scale cultural (including commercial and sporting) events which have a dramatic character, mass popular appeal and international significance’ (Roche, 2000: 1), SMEs – even those that are postponed – present valuable opportunities for understanding certain aspects of one of the largest global crises and tragedies in recent times. More specifically, this article explores how the SME ‘franchise owners’ (Graeff and Knijnik, 2021), the sport governing bodies, such as the International Olympic Committee (IOC) and Union of European Football Associations (UEFA), responded to the pandemic through their public communications and discourses.

In doing so, the article first offers an understanding of how the SME universe entered a standstill between February and March 2020 and this standstill's surrounding discourses. Then, it investigates exactly how sport governing bodies positioned themselves publicly with regard to responding to COVID-19 from within sports. Crucially, these exemplars of public communications remain particularly relevant since sporting bodies ‘must […] communicate with the global public on an ongoing basis to promote the sport, enhance its visibility and attest to its fairness and legitimacy’ (Murray and Pigman, 2014: 1111). Therefore, the communicated responses surrounding SME postponements can assist our understanding of the processes surrounding the postponement decisions and the roles of international sporting bodies in the global crisis.

From a reading of media sources and public communications, this article advances two key arguments. First, that the sporting standstill in February and March 2020 involving, inter alia, the postponement of the giants of the 2020 Olympics in Tokyo and the multi-country 2020 European Football Championship (Euro 2020) reflected the wider transition of COVID-19 from a ‘risk’ to an ‘immediate threat’ (see Domingues, 2020). Second, the article demonstrates how sport governing bodies sought expertise from health fields, including health experts and organisations. In a way, they adopted similar regulatory mechanisms that could be witnessed in the wider political circles in the responses to a pandemic, which did not distinguish between sport and the wider society. Collectively, these arguments are sociologically telling: essentially the staging of an SME links together sport governing bodies, corporations, non-governmental organisations (NGOs) and other institutions (Giulianotti and Brownell, 2012), and these structures were disrupted by the crisis. Further, critical analyses of sports can extend our understanding of wider global issues (such as a pandemic) (Giulianotti and Robertson, 2004) and governance (Włoch, 2012). The insights of this paper underscore these positions in a new context where international organisations were forced to act and respond.

Taken together, the article extends pre-existing literature in three separate ways. First, it chronologically breaks down the dramatic sporting standstill between February and May 2020; the period in which the World Health Organization (WHO, 2020) declared that COVID-19 was a pandemic. Second, the article contributes to the growing scholarship on the powerful nexus between sport and COVID-19, and reflects calls for research on the impacts of COVID-19 in the sports world (Parnell et al., 2020; Rowe, 2020). Finally, this paper offers a sociological understanding of how sport governing bodies – representing the SME ‘franchise owners’ (Graeff and Knijnik, 2021) – publicly communicated their SME-related responses to COVID-19 between February and May 2020. Focusing on the postponed 2020 Olympics and Euro 2020, this paper adds to the pre-existing literature on these global media events. Yet, more distinctively and uniquely, it also offers novel insights that can answer the emerging questions related to the dramatic postponements of the two SMEs.

## Literature review: between risk and threat

This article is concerned with how the ‘risk’ and ‘threat’ of COVID-19 was responded to by sport governing bodies and key actors in the terrain of SMEs between February and May 2020. This represented the period where the futures of several sporting events were decided upon by their relevant administrators and organisers (Tovar, 2020; Weed, 2020). Yet, first, it remains important to unpack COVID-19 as a ‘risk’ and ‘threat’, and the broader state and governmental responses to the pandemic. The new coronavirus was first detected in Wuhan, China, in December 2019 (WHO, 2020). Between December 2019 and February 2020, the infectious disease, COVID-19, caused by the SARS-CoV-2 virus first extended to Asian regions and, eventually, worldwide. Whereas COVID-19 was first characterised as an epidemic, it was confirmed on 11 March, by WHO (2020), that it constituted a pandemic. Ultimately, pandemics and their associated responses warrant sociological analyses – and ‘[a]s a portal, the virus demands that we all think sociologically’ (Matthewman and Huppatz, 2020: 6). Although it remains important to acknowledge that the crisis is a ‘moving target’ that is continuing (Domingues, 2020: 13) at the time of writing.

Infectious disease outbreaks require rapid responses. Indeed, the ‘case of COVID-19 has proved the importance of governments responding swiftly in face of a pandemic to prevent viruses becoming a monstrous agent’ (Zinn, 2020: 1088). Swift responses, ultimately, depend on states and supranational organisations, like WHO, that can influence the political responses and disease governance (Hanreider and Kreuder-Sonnen, 2014). However, responses do not automatically translate into disease elimination – and in this highly unpredictable and uncertain climate, Beck’ s (1992) risk society theory can assist an understanding of the global crisis (Domingues, 2020; Zinn, 2020).

Beck (1992) observed an increasingly globalised world where ‘risks’ became the key drivers for social change. Accordingly, a ‘risk’ refers to ‘a systematic way of dealing with hazards and insecurities induced and introduced by modernity itself’ (Beck, 1992: 21). Beck drew attention to the shift away from an industrial society towards the ‘risk society’. Some of the key pillars here include risks’ time-related and geographical mobilities and disastrous potential. In societies engrossed with risks and uncertainties – and, indeed, mitigating them – and where risks exceed national borderlines and temporal settings, there is also an increased dependence on science and experts. Importantly, in the management of risk, politicians, governments and individuals have come to depend substantially on expert guidance and scientific knowledge (Nygren and Olofsson, 2020). Hence, Beck's ideas may ‘help us make sense of the present [COVID-19] crisis’ (Domingues, 2020: 3), and as Zinn (2020: 1083) submits regarding global risks:

From the early days of technological disasters, through to the financial crisis, international terrorism and climate change, cosmopolitan spaces open up for international collaboration, necessary to successfully manage such global challenges

COVID-19 represents another ‘global challenge’ – which is unselective, transnational (Beck, 1992) and does not ‘respect national boundaries’ (Hanreider and Kreuder-Sonnen, 2014: 335–336). Globally, various public health measures have been implemented by governments, NGOs and individuals (Mann et al., 2020; Mutz and Gerke, 2021). Some of the pandemic responses have included quarantines, social distancing, lockdowns and restrictions on people's movements, including travelling bans and suspensions of public gatherings and social life. Still, despite international collaborative efforts, states have responded *differently* to the pandemic. For instance, the responses to the first outbreak of COVID-19 were marked by the ‘extreme uncertainty of the measures taken by the governments of the various countries to stem the pandemic spread’ (Corsini et al., 2020: 1186). Crucially, however, such measures are not solely implemented to manage the pandemic *risk.* Indeed, Domingues (2020) points to the pandemic's transition from a ‘risk’ to a ‘threat’:

Now we no longer face risk, we are confronted with a *concrete, immediate threat*: the spread of the new coronavirus and Covid-19 disease, costing lives and disrupting collective existence. If risk refers to something possible or likely to happen, a threat is already a moving entity, in this case a specific one: the coronavirus […] A threat hence appears as an immediate danger, a concrete possibility of harm, not a sort of virtual, abstract risk. Threat instantiates risk, we may say (Domingues, 2020: 3, original emphasis)

This transition remains central, and notwithstanding, the need to respond to the risk and concretised threat of COVID-19 has not been confined to political, scientific, health or educational circles. Fundamentally, sports represent a key domain where COVID-19 has had enormous impacts (Parnell et al., 2020) and has required responses from sports' governors.

### Pandemic responses and SMEs

In the sports world, as central here, it is detectable that numerous SMEs have been postponed or cancelled (Weed, 2020). Postponed SMEs include the 2020 Tokyo Olympics, Euro 2020 (originally to be staged across 12 host countries) and the *2020 Copa América* (to be hosted by Argentina and Columbia). Here, it remains imperative to underline that SME postponements or cancellations do represent key responses to COVID-19 made by the distinctive sport governing bodies such as UEFA, IOC or *Fédération Internationale de Football Association* (FIFA). Still, the discourses surrounding the decisions to postpone the mega-events require further investigation in the pandemic's context.

Critically engaging with the SME-related discourses and dynamics can facilitate a disaggregation of sport governing bodies' responses to the pandemic. This remains relevant because studies establish that sport governing bodies are powerful and emerging global actors that govern sport (Boykoff, 2020; Millward, 2017; Włoch, 2012) and ‘set the rules of the game’ (Włoch, 2012: 307) before every SME. For example, according to Boykoff (2011: 42), the IOC may be positioned ‘[s]omewhere between multinational corporation and global institution’, whereas SMEs reveal networked relationships between nation-states, corporations (i.e. mega-event sponsors) and sport governing bodies (Millward, 2017). Ultimately, SMEs are organised by global sport organisations, but *hosted* by nation-states (Włoch, 2020). Besides, the flurry of public/private interests and supranational authority has, of course, become apparent in the responses to the health crisis.

Engagement with sport governing bodies’ responses to COVID-19 remains compatible with the suggestion by Rowe (2020) holding that pandemic sports should be subject to sociological procedures. Since the sports world has not been isolated from COVID-19, it is possible to transplant the discussed ideas of ‘risk’ and ‘threat’ in relation to COVID-19 (Domingues, 2020; Zinn, 2020) into sports' terrain. Ultimately, there is a gap in the literature with regard to how the global ‘risk’ and ‘threat’ of COVID-19 was initially responded to by sport governing bodies and other key actors in the dramatic period where numerous SME's futures were decided upon and discussed. One exception here is the study by Hindman et al. (2021) on the National Basketball League's (NBA) organisational responses to COVID-19. Here, it is demonstrated how the NBA's response was characterised by an acknowledgement of the uncertainty, the desire to operate cautiously whilst also taking risks in the attempt to resume the competition following its suspension in March 2020. Yet, overall, little is known about SME ‘franchise owners’' responses to COVID-19. Here, it is also possible to apply some key premises from Beck's theory to sports and to present-day SMEs. Nonetheless, despite important exceptions (i.e. Cleland, 2019), it argued that: ‘In sport, Beck’s analysis has had surprisingly little impact, possibly because of the limited risk reflexivity within sporting cultures’ (Giulianotti, 2009: 551).

Therefore, this article seeks to examine how some sports governing bodies responded to the pandemic *beyond* merely announcing SME postponements/cancellations, and how these decisions were publicly communicated. This may offer insight into the governance and politics of SMEs and the pandemic. Further, in themselves, postponed mega-events remain interesting to explore since they are extremely rare (Tovar, 2020), generate enormous media interest (Giulianotti and Collison, 2020) and come at huge financial costs (AP News, 2020a). In order to examine sport governing bodies' responses to COVID-19, this article will first explore how the SME world – much like the rest of the world – entered a mode of ‘standstill’ between February and March 2020. Then, it sets out to examine how sport governing bodies, subsequently, publicly responded to – and positioned themselves – with regard to resolving the emerging threat.

## Method and theoretical position

The article focuses on the postponements of the 2020 Summer Olympics (owned by IOC) and Euro 2020 (owned by European football’s governing body, UEFA). The selection of these specific SMEs is justified by the fact that, first, these are among the most popular SMEs globally and traditionally have attracted large numbers of visitors, whilst corresponding with Roche’s (2000) aforementioned mega-event definition. Second, and crucially, both these SMEs were originally scheduled between June and August 2020, whereas these mega-events’ futures received broad media coverage – demonstrating the widespread interest in these events, and the general sports-COVID nexus (Giulianotti and Collison, 2020).

The article adapts a digital qualitative research approach and draws its data from media quotes and interview material *within* media sources (collected through Google News) and official statements/communications from sport governing bodies' official websites. The selection of this type of data source is explained by three key reasons. First, the media was among the key sites where the SME's doubtful futures and postponements were commented-upon or discussed – even in a global crisis with limited live sports (Giulianotti and Collison, 2020). Furthermore, ‘[w]ebsites have become major portals for international sporting bodies to communicate about their aims and the aims of the sport’ (Murray and Pigman, 2014: 1111). Second, the turn towards secondary sources was made in the context of the unobtrusiveness of this approach during a time where social distancing measures, understandably, impacted methodological alternatives. Third, the strengths of electronic media sources are confirmed in similar studies investigating the discursive framing of various socio-political issues around SMEs (Atkinson and Young, 2012).

Upon data collection, targeted searches were performed for media articles that considered or mentioned the relevant events, which contained interview materials from key actors/organisations around the mentioned SMEs. Following Atkinson and Young (2012: 290), there was no rigid selection criteria that guided the sampling strategy, which may be best described as a ‘blend of purposive and convenience sampling’. This was then operationalised by using the following terms to inform the targeted searches: COVID-19, coronavirus, Euro 2020, Olympics, IOC and UEFA. Upon sampling, however, the inclusion criteria were that the media sources were written in English language, published between 1 February and 31 May 2020, addressed or considered the relevant SME's futures (in COVID-19’s context), and/or included quotes/statements by key actors/organisations of the two SMEs. Consequently, media sources solely mentioning the 2020 Olympics or Euro 2020 in passing, or published before/after the timeframe, were excluded from the final sample, which meant that, overall, 82 electronic media sources were sampled together with 26 official communications/announcements released by UEFA, IOC, or relevant sporting bodies. The specific timeframe of these sources (1 February–31 May 2020) was selected because this represented the period in which the relevant events' futures were discussed, acted upon, and where the *immediate* postponement-related aftermath occurred. In the beginning of March 2020, the number of global COVID-19 cases surpassed 100,000 (WHO, 2020) and it was when COVID-19's impact ‘became clearer during February and March 2020, sports event hosts and administrators began to consider whether their events should be postponed or cancelled’ (Weed, 2020: 82). Then, as Bandyopadhyay (2021: 1) notes, ‘the gradual resumption of games and tournaments from May 2020 onwards began to represent the early impressions and initial responses to the pandemic’. In this period, a vaccine was not yet developed. Therefore, although this study's 4-month timeframe appears limited, this period proved extremely dramatic in the sports world.

Importantly, newspaper articles should be approached with some caution given the possible journalistic bias, newspapers’ profitable aims or the potential lack of context. Notwithstanding, following Millward (2017), the study is concerned with the quotes/statements from key individuals/organisations available *within* the media sources. Therefore, the unit of analysis is *not* the journalistic accounts of the postponed SMEs. Similar to Millward's study of the 2022 FIFA Men's World Cup build-up, the units of analysis are the public statements and quotes *within* the media sources. Still, the idea of ‘fantasy documents’ (Boyle and Haggerty, 2012) may be useful in this context. Published as reports, statements or policy documents, these articulations may be issued by sport governing bodies in times of uncertainty. They are ‘formulated specifically to address public anxieties’ (Boyle and Haggerty, 2012: 253) and to generate reassurances. Whilst this implies that caution must be exercised concerning a possible non-correspondence between *public* rhetoric and the *actual* realities, it would concurrently ‘be too cynical to regard such documents as outright fabrications’ (Boyle and Haggerty, 2012: 252). And principally, this article remains concerned with how pandemic responses were *publicly* communicated.

### Frame analysis

The collected statements/quotes were manually analysed with a frame analysis technique developed by theorist Goffman (1974). Essentially, Goffman's influential work was principally concerned with social interaction contexts, and in *Frame Analysis*, Goffman underlined the importance of the organisation of experience; especially how ‘individuals organize their experiences into meaningful activities and settle on a clear definition of *their* reality’ (Millward, 2017: 762). The relevant social actors' definitions of specific situations remain central here; and in this regard, Goffman (1974) observed how specific segments of discourses were more heavily weighted than others, and how discourses proceed to impact how specific situations are described.

Frames are accordingly defined as the ‘principles of organization which govern events – at least social ones – and our subjective involvement in them’ (Goffman, 1974: 10–11). Therefore, frames can serve to organise and interpret experiences of the social world and its realities. However, importantly, frame analysis techniques can also be applied to the discourses of sport governing bodies. Here, such approaches aid understandings of how sporting bodies – representing social actors – frame specific situations or developments (see Millward, 2017).

Hence, the collected statements and media sources were frame analysed according to two keyframes corresponding with my research aims. First, how COVID-19 was discussed and described by sport governing bodies as the future of the 2020 Olympics and Euro 2020 became increasingly doubtful. This would then allow for an understanding of how the unfolding realities of the pandemic were experienced and defined by the sport governing bodies. Second, how the issue of resolving the global crisis was framed by the sport governing bodies in relation to the relevant SMEs.

## Tracing the sporting standstill

The cancelled or postponed SMEs, of course, represented crucial and highly visible responses to the pandemic from the sports world. This section breaks down and provides a narrative of the period in which sports gradually entered a standstill (primarily between February and March 2020). This is complemented and contextualised by the framed discourses from sport governing bodies in this period. As argued, the initial responses and emerging discourses occurred alongside the wider (see Domingues, 2020) and indeed sporting-specific concretisation of COVID-19 as a threat.

For context, throughout January and February 2020, COVID-19 was still considered an epidemic and it remained unclear exactly what impact the crisis would have on SMEs and the rest of the world, although early reports suggested that the infectious disease *could* impact the 2020 Olympics (Reuters, 2020). In China – where the virus was first detected – the *Chinese Super League* was suspended on 30 January 2020 (Tovar, 2020). In Europe, however, sporting events were not suspended at first (Corsini et al., 2020). Until late February 2020, Euro 2020 seemed to be staged as planned, until indications of what was unfolding emerged from Italy, as *Serie A* games began to take place behind ‘closed doors’, since Italy (one of Euro 2020’s hosts), was affected earlier than other countries by the virus (Corsini et al., 2020). A Rugby Six Nations match, to be staged in Dublin on 7th March, between Ireland and Italy, was also postponed over ‘health concerns’ on 26 February (BBC, 2020a). Not long after, *The Independent* (2020) reported that the virus had forced UEFA into ‘crisis talks’ concerning Euro 2020. As UEFA Vice President, Michele Uva, commented: ‘We are monitoring country by country, and football must follow the orders of the individual countries. The sporting path will only be closed if the situation gets worse’ (quoted in *The Independent*, 2020). Interestingly, here one may see how Euro 2020's hosting style, involving 12 different European countries, was framed as a particular issue (‘monitoring country by country’).

On 3 March, reports again emerged over Euro 2020's future (*The Guardian*, 2020a) and UEFA President Aleksander Čeferin acknowledged that the coronavirus added to the security-related concerns and political obstacles that usually surround contemporary SMEs such as ‘terrorism’ (Cleland, 2019), crime or ‘hooliganism’:

We are dealing with it and we are confident we can deal with it […] You don't know how many big concerns we have: we have security concerns, political instability and one is also the virus. Let's try to be optimistic, not think about dark scenarios – there's time for that later (quoted in *The Guardian*, 2020a)

Meanwhile, in a different UEFA tournament – the Champions League – the fixtures on 10 March were characterised by inconsistency, with the situation and responses differing between European countries. In Spain, Valencia–Atalanta took place behind closed doors, whereas RB Leipzig–Tottenham was staged in front of a full crowd in Germany. The next day, when WHO confirmed that COVID-19 was a pandemic (Corsini et al., 2020), Paris Saint German-Borussia Dortmund took place behind closed doors, whereas Liverpool–Atletico Madrid was staged with 52,000 fans inside the stadium (Bandyopadhyay, 2021) – demonstrating the country-specific measures apparent vis-à-vis sporting events. In a way, key turning points were the WHO's assessments, COVID-19 becoming a pandemic, and reports of infected athletes and managers (BBC, 2020b), as several domestic leagues were suspended between 12 and 13 March (see Tovar, 2020).

The unfolding situation and the suspension of domestic leagues called for action in relation to the 2020 Olympics and Euro 2020. For example, UEFA announced on 12 March that they invited to a stakeholder meeting, following the ‘ongoing developments in the spread of COVID-19 across Europe and the changing analysis of the World Health Organization,’ which would also involve discussions about Euro 2020 (UEFA, 2020a). On 13 March, football's international governing body, FIFA, issued a statement that set the tone for the forthcoming week. FIFA (2020) recommended that all international games scheduled for March and April should be postponed until they could be staged in a ‘safe and secure environment.’

Then, on 17 March 2020, UEFA confirmed that, following a decision by UEFA's Executive Committee, Euro 2020 had been postponed to 11 June–11 July 2021. The announcement stated that: ‘The health of all those involved in the game is the priority, as well as to avoid placing any unnecessary pressure on national public services involved in staging matches’ (UEFA, 2020b). With the planned staging of Euro 2020 becoming incompatible with health and safety, Čeferin also commented that:

We are at the helm of a sport that vast numbers of people live and breathe that has been laid low by *this invisible and fast-moving opponent.* It is at times like these that the football community needs to show responsibility, unity, solidarity and altruism (quoted in UEFA, 2020b, emphasis added)

Ultimately, this quote reveals the virus's acceleration-based characteristic as a ‘fast-moving opponent’. However, as Weed (2020: 82) writes, there was still – as late as 19 March 2020 – an insistence around the Olympics that ‘it was too early to decide whether to cancel the Games’. As an IOC (2020a) communique released on 18 March stated: ‘there is no need for any drastic decisions at this stage’. Such a stance was criticised and, as Giulianotti and Collison (2020: 6) highlight, the ‘IOC leadership attracted further protests from many athletes and some sport federations for delaying the seemingly unavoidable decision, to postpone the Tokyo 2020 Olympics’. With the rapidly changing circumstances and some nations declaring their intention not to participate – should the Olympics proceed as planned – the 2020 Olympics were eventually postponed on 24 March. As the Tokyo Organising Committee of the Olympic and Paralympic Games (Tokyo 2020) announced:

In the present circumstances and based on the information provided by the WHO today, the IOC President and the Prime Minister of Japan have concluded that the Games of the XXXII Olympiad in Tokyo must be rescheduled to a date beyond 2020 but not later than summer 2021, to safeguard the health of the athletes, everybody involved in the Olympic Games and the international community (Tokyo 2020, 2020)

Similar to UEFA's statement, one can observe that a situation is framed where the health and safety of individuals (the ‘athletes’ and ‘everybody involved’) had to be provided protection. This connects with Mann et al. (2020: 1071), who note that ‘the rhetoric emerging from international sporting organisations, such as the International Olympic Committee (IOC), has emphasised the importance of protecting athlete health’. On 25 March, when commenting on the factors contributing to IOC's altered stance, IOC President Thomas Bach commented that this was because of ‘developments with the dynamic spreading of the coronavirus’ and the alarming information on the accelerated virus spread (quoted in IOC, 2020b). With the Olympics rescheduled to between 23 July and 8 August 2021 (Constandt and Willem, 2020), the then-head of Japan's Olympic organising committee, Yoshiro Mori, also spoke of an unprecedented situation: ‘In the past, when there were such problems, like wartime, it has been cancelled. This time, we are fighting an invisible enemy’ (quoted in Sky News, 2020).

The above breakdown of events and the discourses surrounding the eventual postponements reveal several important processes. First, these SME postponements must be considered historical moments within sports' social history, where postponements are rare (Tovar, 2020) and testing for stakeholders. As Constandt and Willem (2020: 53) put it, ‘a postponement of such size is unseen in the history of the modern Olympic Games’. Moreover, the gradual change of stance is remarkable and illustrates a shift from a publicly articulated view maintaining that the SMEs, possibly, could proceed – to the postponement decisions, in order to prioritise and safeguard health and safety.

In a way, the above discourses can demonstrate the aforementioned transition of COVID-19 from a ‘risk’ towards an ‘immediate threat’, whereby a threat is characterised by its immediacy and concrete realities (Domingues, 2020). Whereas this does not mean the pandemic was not causing harm and deaths before this concretisation, one may see that the pandemic transitions from being *one* of the several risks that mega-events are exposed to, and must contemplate, towards becoming *the* main threat. As Domingues writes, this shift was ‘necessitating changes in how the nation-state intervenes and how global health administration – particularly at a political level – will possibly change’ (Domingues, 2020: 13). This happens in parallel with a situation where the crisis unfolds rapidly and where uncertainty and harm are defining and present features. Arguably, this important shift, when it occurs in the wider society, has reflexive consequences in the terrain of sport, which is neither isolated nor exempt.

This meant that SME hosts and key actors, as shown, had to intervene and respond by deciding to postpone their SMEs, as the cases of Euro 2020 and the 2020 Olympics collectively exemplify. It is also worth noticing what the next section explains in detail. Namely, the framed turn towards health experts beyond sports' realm. This is, for example, demonstrated by the changing assessments of WHO which, as framed by UEFA and IOC above, would partly inform the eventual decisions to postpone the relevant mega-events originally scheduled between June and August 2020. Hence, although SME postponements or cancellations represented the most obvious and visible initial responses to COVID-19 vis-à-vis SMEs, this section disassembles – in a chronological manner – the framed discourses from these events' key actors. Unpacking these discourses simultaneously gives a glimpse of what seemingly informed the postponement-related responses throughout late-February and March 2020.

## Finding expertise beyond sports

Pandemics require immediate responses. Indeed, Hanreider and Kreuder-Sonnen (2014: 336) note that pandemics are among the ‘prototypical crisis scenarios in which high-speed decisionmaking and rapid political interventions are seen to be needed’. This need, to urgently respond, and eventually overcome the health crisis, can also be situated in sports. Therefore, this section maps out the prognostically framed outcomes of COVID-19. It explores how sport governing bodies framed the issues of resolving the global crisis and how they publicly communicated their own position vis-à-vis responding to COVID-19 beyond SME postponements and in relation to safely staging the rescheduled events.

Although the discussed postponements, in themselves, represented key responses made by sport governing bodies, these did not serve to resolve the pandemic more broadly. Instead, the rescheduling of SMEs could help containi the outbreaks in the overarching climates of uncertainty. In the face of a threat that, essentially, *did not* distinguish between SMEs and the rest of the society, it is argued that the below examples demonstrate how sport governing bodies looked towards knowledge and guidance from health experts and international health organisations. Consequently, scientific progress and health expertise became firmly embedded into the discourses of how SMEs could safely return.

This could, however, be observed even *before* the SMEs were postponed. On 3 March 2020, IOC (2020c) could report that they would ‘continue to follow the advice of WHO’, and that: ‘A joint task force had already been created in mid-February, involving the IOC, Tokyo 2020, the host city of Tokyo, the government of Japan and the World Health Organization (WHO)’. One could also see how the ‘changing analysis of the World Health Organization’ (UEFA, 2020a) and ‘information provided by the WHO’ (Tokyo, 2020) were framed earlier in the context of the decisions to postpone the respective SMEs. Further, in light of Euro 2020's postponement (17 March 2020), UEFA together with the European Club Association, European Leagues and FIFPRO Europe signed a resolution on ‘how European football should react to the challenges created by the COVID-19 pandemic’ (UEFA, 2020c). This coordinated response involved a contingency plan which ‘[took] into consideration the advice of international health experts as well as the restrictive orders issued by national governments and local authorities (UEFA, 2020c)’. Already here, the distinctive turn towards health expertise becomes apparent.

A similar framing was also evident in the communication of IOC (2020d) on 6 May 2020, where WHO was described as ‘instrumental’ by giving IOC ‘real-time information’ prior to the Olympic postponement. Further, it confirmed that: ‘WHO continues to advise the IOC and the Tokyo 2020 Organising Committee on how to ensure that the Olympic Games will take place in a safe environment for all those involved’ (IOC, 2020d). WHO is framed by IOC as the ‘instrumental’ actor from which the necessary expert advice could be obtained in the build-up for a safe staging of the rescheduled Olympics. In the context of disease governance, WHO can be considered a global emergency governor that can define crises and emergencies, provide or formulate policy guidelines, and guide political responses (Hanreider and Kreuder-Sonnen, 2014). As this evidence suggests, this extended into the terrain of sport, and a similar framing becomes apparent in another statement issued two days later:

We will follow the risk management and mitigation measures set out by the World Health Organization (WHO) for mass gatherings […] We will continue to follow the principle that has driven all our decisions so far, which is to organise Olympic Games only in a safe environment for all people involved (IOC, 2020e).

In a way, the framed efforts to provide a ‘safe environment’ reflect the broader public reliance in and dependence on expert systems (Nygren and Olofsson, 2020) and can illuminate the politics of knowledge and hierarchies of relevant expertise that emerged throughout the COVID-19 pandemic. Since overcoming COVID-19 largely remains a scientific question, sport governing bodies seemingly adopted a position where they adapted to the guidelines from the discursively framed health experts, as central to the wider pandemic responses. However, in a way, those framed health experts still depended on sport governing bodies to act. On 18 March, when the 2020 Olympics' future was still not decided upon, WHO spokesperson, Tarik Jasarevic, stated that:

It is not the role of WHO to call off or not call off any type of events […] As each international mass gathering is different, the factors to consider when determining if the event should be cancelled may also differ. Any decision to change a planned international gathering should be based on a careful assessment of the risks and how they can be managed, and the level of event planning (quoted in *The Guardian*, 2020b).

The above statements not only demonstrate the increased centrality of international health experts – who remain externally placed in relation to sports – to sport governing bodies' decision-making and attempts to ensure the safe rescheduling of SMEs. They also illustrate how health organisations like WHO still depended on sporting bodies to take action ('call off or not call off any type of events'). Although, the main point here is that sport governing bodies seemingly were influenced by the analysis, directives and assessments produced by actors on the *outside* of sports.

This, in itself, remains unsurprising. Yet, this is a broader trend that can be identified at the state, governmental and institutional levels. When global crises occur, uncertainty looms large and urgent decision-making is necessary, ‘turning to IOs [international organisations] is a natural choice because of both their centralisation and their expertise’ (Hanreider and Kreuder-Sonnen, 2014: 336). Notwithstanding, the point made here is that it was not merely governments nor authorities that looked towards health experts in the state of crisis. Seemingly, such a stance was consciously replicated by sport governing bodies too. Thus, as sport governing bodies arrived at their decisions to postpone SMEs – and eventually began planning for the rescheduled mega-events, the decision-making and planning were seemingly informed by the guidelines emanating from health experts that were externally found: outside the sphere of sports. In part, this can help us understand the SME ‘franchise owners’ communicated responses to COVID-19.

The public communications above remain telling since they suggest a turn towards expert voices and scientific knowledge in the period of global crisis. Despite this, however, it is clear that uncertainty levels still existed and that there were no guarantees that the pandemic would be ‘under control’ by the Olympics' new dates (Constandt and Willem, 2020: 54). Indeed, even at the time of writing, less than three months before the 2020 Olympics are due to commence, extreme uncertainty still surrounds this Olympic edition (*The Independent*, 2021; Shimizu et al., 2021). Meanwhile, in March 2021, international spectators were banned from travelling to attend the Games over COVID-19 concerns (CNN, 2021). Furthermore, the scientific debate consists of a ‘heterogeneous supply of scientific interpretations’ (Beck, 1992: 157) and COVID-19 is surrounded by ‘contested knowledge claims’ (Wardman and Lofstedt, 2020: 834). One can see hints towards this reality, as the UEFA President Čeferin, when commenting on the return of ‘normality’ and European football, acknowledged that: ‘Even the expert doctors don’t know when this [COVID-19] will finish […] The more we will respect that [COVID restrictions], the faster the crisis will finish’ (quoted in AP News, 2020b).

In the risk society context, such acknowledgement illustrates the unpredictability and uncertainty that endured despite following the paths of health experts. Overall, this feeds into this section, which demonstrates sports bodies' conscious turn towards guidelines and recommendations from health experts and international organisations. Whilst such a move, in isolation, may seem unsurprising, these exemplars display, within the specific timeframe, how sport governing bodies' responses to the unfolding crisis, in distinctive ways, reflected the broader political and governmental responses to the pandemic. This saw scientific knowledge and health specialists becoming embedded into the planning and safe delivery of the rescheduled SMEs.

## Conclusion

For Roche (2000: 235), mega-events occupy sociologically important positions as ‘intergenerational cultural reference points’ that leave memorable and enduring marks on the social calendars of modern societies. Mega-events, Roche argues, are some of modern society's great ‘parades’ and ‘shows’ (Roche, 2000: 1). Against this background, it represented defining turning points when ‘the show’ could not go on, and numerous mega-events originally due to be staged in 2020 were postponed because of the COVID-19 pandemic. This included two SMEs with global profiles, media reach, and popularity: the 2020 Olympics and Euro 2020.

In the case of these SMEs, this article has explored how the sport governing bodies – who organise and administrate the mentioned SMEs – responded to the pandemic through their public communications. This remains of particular relevance since sport governing bodies are powerful, influential and truly global actors (Graeff and Knijnik, 2021; Millward, 2017; Włoch, 2012). In global societies, SMEs form time-specific and networked spaces wherein states (for example, host countries) and non-state actors, like UEFA, FIFA or IOC, enter what Włoch (2020: 46) call ‘intensive interest-driven interactions’. Essentially, these networked structures were impacted by the global crisis. Therefore, this article offers an understanding of supranational organisations' roles, discourses and responses in a global crisis, drawing from a corpus of media sources and sporting organisations' public communications.

This article argues that the sporting ‘standstill’ (the period wherein Euro 2020 and the Olympics were postponed for a year), occurring primarily between late February and March 2020, illustrated how the ‘risk’ of COVID-19 became concretised as an ‘immediate threat’ (Domingues, 2020) requiring intervention in world sport. Further, the article argues that sport governing bodies, through their discourses, emphasised their turn towards externalised expertise in the form of health experts and international organisations, which could provide guidance in the endeavour of responding to and, eventually, trying to resolve the global crisis. Looking to existing literature, such a manoeuvres can be contextualised by the expert dependence and trust which influence risk management and mitigation in uncertainty-loaded risk societies (Beck, 1992).

By synthesising the above arguments, it can be argued that the apparatuses of governance surrounding SMEs resembled the broader regulatory mechanisms of how the pandemic crisis was responded to on a political level. This included decision-making based upon ‘expert authorities’ assessments' (Nygren and Olofsson, 2020: 1032), as with COVID-19, ‘we face an ambivalent situation when the constant presence of risk and uncertainty increases our daily dependence on expertise’ (Nygren and Olofsson, 2020: 1035). Given the pandemic's global scope – both as a risk and threat – this study's arguments can thus support the overarching suggestions that analyses of global issues *in* sport can assist a more mainstream understanding of those same global issues (Giulianotti and Robertson, 2004), though such a position acquires a set of new meanings in the pandemic sport context.

Taken together, this article makes a threefold academic contribution to the sociology of sport and SME studies. First, it disaggregates the discourses, processes and communications related to the sporting standstill between February and May 2020. This was a truly exceptional period where the futures of several SMEs were acted upon by their owners and organisers. This period could be approached as a particularly critical time in sports' social history. Second, as called for (Parnell et al., 2020; Rowe, 2020), the article makes important advances towards a sociological understanding of one aspect of the relationship between sports and COVID-19. Essentially, the ‘pandemic had an extraordinary impact on world sport’ (Giulianotti and Collison, 2020: 1) – and this underscores the importance of critically exploring the sport/pandemic intersection. Third, this article extends our knowledge of two important SMEs with prolonged timelines: the 2020 Olympics and Euro 2020 (Boykoff, 2020; Constandt and Willem, 2020; Hutchins and Andrejevcic, 2021; Lee Ludvigsen, 2021). In themselves, these postponed mega-events are likely to attract more academic attention as their new dates approach in time. Notwithstanding, some limitations of this article should be highlighted. The exploratory study has examined the relationship between SMEs and COVID-19 – and maybe is it too early to reach firm conclusions about ‘something which is still underway’ (Matthewman and Huppatz, 2020: 680). The article also focuses specifically on the unfolding period between February and May 2020. Another caveat is that the paper predominantly focuses on merely two postponed SMEs and the emerging discourses from these respective SMEs' franchise owners. This limits the possibility for making any generalisable claims, yet underlines the need for research on other SMEs postponed/cancelled throughout 2020 and 2021.

COVID-19 has added new elements to the securitised consumption surrounding every SME, namely public health and hygiene (Hutchins and Andrejevcic, 2021). Future research should hence consider the emerging discourses surrounding ‘health’ and ‘safeguarding’ – and their meanings – as the rescheduled events approach and/or are staged. This should be done in relation to attendees, local residents and athletes (see Mann et al., 2020), especially so, should there be an outbreak of the virus before/during upcoming mega-events. As Shimizu et al. (2021) remind us, serious safety-related concerns still exist ahead of the upcoming 2020 Olympics despite the aforementioned exclusion of international spectators in Tokyo. Then, it also remains to be seen how the pandemic and sport governing bodies' management of the crisis have impacted the public opinion on SMEs. For example, at the time of writing, there is widespread public opposition in Japan to the staging of the 2020 Olympics in the summer of 2021 (*The Independent*, 2021). Sport governing bodies' positions and responses may also have impacted anti-Olympic activism (see Boykoff, 2020) or how spectators consider their personal safety when visiting sports venues (Cleland, 2019). Moving forward, exploring such broad questions can advance our understandings of how COVID-19 has impacted and reshaped global sports' or SME's positions in modern society.

## References

[bibr1-10126902211020169] AP News (2020b) Q&A: UEFA president Čeferin on moving Euro to 2021. AP News, 17 March. Available at: https://apnews.com/article/f670b35d61f16c4ca8394716a404a639 (accessed 21 June 2020).

[bibr2-10126902211020169] AP News (2020a) Tokyo Olympics delay costs may reach $2.8 billion. AP News, 4 December. Available at: https://apnews.com/article/national-governments-tokyo-coronavirus-pandemic-2020-tokyo-olympics-japan-089b35abee216b46c8fe39993972fcfb (accessed 9 January 2021).

[bibr3-10126902211020169] Atkinson M Young K (2012) Shadowed by the corpse of war: Sport spectacles and the spirit of terrorism. International Review for the Sociology of Sport 47(3): 286–306.

[bibr4-10126902211020169] Bandyopadhyay K (2021) Introduction: COVID-19 and the soccer world. Soccer & Society 22(1–2): 1–7.

[bibr5-10126902211020169] BBC (2020b) Coronavirus: Chelsea’s callum hudson-odoi and arsenal boss Mikel Arteta test positive. BBC, 13 March. Available at: https://www.bbc.co.uk/sport/football/51867854 (accessed 2 April 2020).

[bibr6-10126902211020169] BBC (2020a) Coronavirus: Ireland v Italy Six Nations games postponed over health concerns. BBC, 26 February. Available at: https://www.bbc.co.uk/sport/rugby-union/51641149 (accessed 28 February 2021).

[bibr7-10126902211020169] Beck U (1992) Risk Society: Towards a New Modernity. London: Sage.

[bibr8-10126902211020169] Boykoff J (2011) The anti-Olympics. New Left Review 67(1): 41–59.

[bibr9-10126902211020169] Boykoff J (2020) Nolympians: Inside the Fight Against Capitalist Mega-Sports in Los Angeles, Tokyo and Beyond. Winnipeg: Fernwood.

[bibr10-10126902211020169] Boyle P Haggerty KD (2012) Planning for the worst: Risk, uncertainty and the Olympic Games. British Journal of Sociology 63(2): 241–259. 10.1111/j.1468-4446.2012.01408.x 22670646

[bibr11-10126902211020169] Cleland J (2019) Sports fandom in the risk society: Analyzing perceptions and experiences of risk, security and terrorism at elite sports events. Sociology of Sport Journal 36(2): 144–151.

[bibr12-10126902211020169] CNN (2021) International spectators will be refused entry into Japan for Tokyo 2020. CNN. Available at: https://edition.cnn.com/2021/03/20/sport/tokyo-2020-announces-ban-for-foreign-spectators-spt-intl/index.html (accessed 28 April 2020).

[bibr13-10126902211020169] Constandt B Willem A (2020) Hosting the Olympics in times of a pandemic: Historical insights from Antwerp 1920. Leisure Sciences 43(1-2): 50–55.

[bibr14-10126902211020169] Corsini A Bisciotti GN Eirale C, et al. (2020) Football cannot restart soon during the COVID-19 emergency! A critical perspective from the Italian experience and a call for action. British Journal of Sports Medicine 54(20): 1186–1187. 32209554 10.1136/bjsports-2020-102306

[bibr15-10126902211020169] Domingues JM (2020) From global risk to global threat: State capabilities and modernity in times of coronavirus. Current Sociology: 1–18.

[bibr16-10126902211020169] FIFA (2020) FIFA Statement 13 Mar 2020. FIFA. Available at: https://www.fifa.com/who-we-are/news/fifa-statement-x8681 (accessed 27 March 2020).

[bibr17-10126902211020169] Giulianotti R (2009) Risk and sport: An analysis of sociological theories and research agendas. Sociology of Sport Journal 26(4): 540–556.

[bibr18-10126902211020169] Giulianotti R Brownell S (2012) Olympic and world sport: Making transnational society? The British Journal of Sociology 63: 199–215. 22670644 10.1111/j.1468-4446.2012.01406.x

[bibr19-10126902211020169] Giulianotti R Collison H (2020) Sport and the covid-19 pandemic: A structuralist analysis of key themes in the UK mass media. Frontiers in Sports and Active Living 2: 1–10. 10.3389/fspor.2020.578472 PMC7739720 33345143

[bibr20-10126902211020169] Giulianotti R Robertson R (2004) The globalization of football: A study in the glocalization of the ‘serious life’. The British Journal of Sociology 55(4): 545–568. 15663424 10.1111/j.1468-4446.2004.00037.x

[bibr21-10126902211020169] Goffman E (1974) Frame Analysis: An Essay on the Organization of Experience. Plymouth: Penguin.

[bibr22-10126902211020169] Graeff B Knijnik J (2021) If things go south: The renewed policy of sport mega events allocation and its implications for future research. International Review for the Sociology of Sport: 1–18.

[bibr23-10126902211020169] Hanreider T Kreuder-Sonnen C (2014) WHO Decides on the exception? Securitization and emergency governance in global health. Security Dialogue 45(4): 331–348.

[bibr24-10126902211020169] Hindman LC Nefertiti AW Kwame JAA (2021) Bounded rationality or bounded morality? The national basketball association response to COVID-19. European Sport Management Quarterly: 1–17.

[bibr25-10126902211020169] Hutchins B Andrejevcic M (2021) Olympian surveillance: Sports stadiums and the normalization of biometric monitoring. International Journal of Communication 15: 363–382.

[bibr26-10126902211020169] IOC (2020a) Communique from the International Olympic Committee (IOC) regarding the Olympic Games Tokyo 2020. IOC, 17 March. Available at: https://www.olympic.org/news/communique-from-the-international-olympic-committee-ioc-regarding-the-olympic-games-tokyo-2020 (accessed 28 April 2020).

[bibr30-10126902211020169] IOC (2020b) IOC President: “It will require everybody’s effort to make these games a symbol of hope”. IOC, 25 March. Available at: https://www.olympic.org/news/ioc-president-it-will-require-everybody-s-efforts-to-make-these-games-a-symbol-of-hope (accessed 28 April 2020).

[bibr29-10126902211020169] IOC (2020c) IOC Executive board statement on the coronavirus (COVID-19 and the Olympic games Tokyo 2020. IOC, 3 March. Available at: https://www.olympic.org/news/ioc-executive-board-statement-on-the-coronavirus-covid-19-and-the-olympic-games-tokyo-2020 (accessed 12 January 2021).

[bibr28-10126902211020169] IOC (2020d) IOC And sports events organisers to continue to benefit from WHO guidance on mass gatherings. IOC, 6 May. Available at: https://www.olympic.org/news/ioc-and-sports-events-organisers-to-continue-to-benefit-from-who-guidance-on-mass-gatherings (accessed 12 January 2021).

[bibr27-10126902211020169] IOC (2020e) Frequently asked questions about the olympic games Tokyo 2020. IOC, 8 May. Available at: https://www.olympic.org/news/ioc/tokyo-2020-q-a (accessed 12 January 2021).

[bibr31-10126902211020169] Lee Ludvigsen JA (2021) Between security and festivity: The case of fan zones. *International Review for the Sociology of Sport* 56(2): 233–251.

[bibr32-10126902211020169] Mann RH Clift BC Boykoff J, et al. (2020) Athletes as community; athletes in community: Covid-19, sporting mega-events and athlete health protection. British Journal of Sports Medicine 54(18): 1071–1072. 32303522 10.1136/bjsports-2020-102433 PMC7497562

[bibr33-10126902211020169] Matthewman S Huppatz K (2020) A sociology of covid-19. Journal of Sociology 56(4): 675–683.

[bibr34-10126902211020169] Millward P (2017) World Cup 2022 and Qatar’s construction projects: Relational power in networks and relational responsibilities to migrant workers. Current Sociology 65(5): 756–776.

[bibr35-10126902211020169] Murray S Pigman GA (2014) Mapping the relationship between international sport and diplomacy. Sport in Society 17(9): 1098–1118.

[bibr36-10126902211020169] Mutz M Gerke M (2021) Sport and exercise in times of self-quarantine: How Germans changed their behaviour at the beginning of the covid-19 pandemic. International Review for the Sociology of Sport 56(3): 305–316.

[bibr37-10126902211020169] Nygren KG Olofsson A (2020) Managing the covid-19 pandemic through individual responsibility: The consequences of a world risk society and enhanced ethopolitics. Journal of Risk Research 23(7-8): 1031–1035.

[bibr38-10126902211020169] Parnell D Widdop P Bond A, et al. (2020) COVID-19, networks and sport. Managing Sport and Leisure: 1–7.

[bibr39-10126902211020169] Reuters (2020). Japan And IOC deny that olympics will be cancelled. Reuters, January 21. Available at: https://www.reuters.com/article/us-olympics-2020-cancellation-idUSKBN29Q34X (accessed 9 January 2021).

[bibr40-10126902211020169] Roche M (2000) Mega-Events and Modernity: Olympics and Expos in the Growth of Global Culture. London: Routledge.

[bibr41-10126902211020169] Rowe D (2020) Subjecting pandemic sport to a sociological procedure. Journal of Sociology 56(4): 704–713.

[bibr42-10126902211020169] Shimizu K Sridhar D Taniguchi K, et al. (2021) Reconsider this summer’s Olympic and Paralympic games. British Medical Journal: 1–2. 10.1136/bmj.n962 33853866

[bibr43-10126902211020169] Sky News (2020) Coronavirus: Tokyo Olympics to be cancelled altogether if COVID-19 vaccine not found soon. Sky News, 28 April. Available at: https://news.sky.com/story/coronavirus-tokyo-olympics-to-be-cancelled-if-covid-19-vaccine-not-found-soon-japans-chief-medic-11979946.

[bibr44-10126902211020169] The Guardian (2020b) Olympics must be delayed to ensure “equal” competition. The Guardian, 18 March. Available at: https://www.theguardian.com/sport/2020/mar/18/ioc-counting-on-solidarity-of-athletes-as-dissent-grows-over-2020-olympics.

[bibr45-10126902211020169] The Guardian (2020a) UEFA’s Ceferin warns against fixating on ‘dark scenarios’ over coronavirus threat. The Guardian, 3 March. Available at: https://www.theguardian.com/football/2020/mar/03/uefa-congress-ceferin-coronavirus-dark-scenarios.

[bibr46-10126902211020169] The Independent (2020) Coronavirus forcing UEFA into crisis talks as Euro 2020 fears mount. The Independent, 28 February. Available at: https://www.independent.co.uk/sport/football/international/euro-2020-coronavirus-news-postponed-uefa-latest-a9365166.html.

[bibr47-10126902211020169] The Independent (2021) Olympics: 80 per cent of Japanese want Tokyo Games cancelled or delayed. The Independent, 10 January. Available at: https://www.independent.co.uk/news/world/asia/olympics-pandemic-tokyo-japan-survey-b1785077.html (accessed 28 April 2020).

[bibr48-10126902211020169] Tokyo 2020 (2020) Olympic games postponed to 2021. Tokyo 2020, 24 March. Available at: https://tokyo2020.org/en/news/joint-statement-from-international-olympic-committee-and-tokyo2020#:∼:text=In%20the%20present%20circumstances%20and,health%20of%20the%20athletes%2C%20everybody.

[bibr49-10126902211020169] Tovar J (2020) Soccer, World War II and coronavirus: A comparative analysis of how the sport shut down. Soccer & Society 22(1–2): 66–74.

[bibr50-10126902211020169] UEFA (2020c) Resolution of the European football family on a coordinated response to the impact of the COVID-19 on competitions. UEFA, 17 March. Available at: https://www.uefa.com/insideuefa/about-uefa/news/newsid=2641077.html (accessed 27 May 2020).

[bibr51-10126902211020169] UEFA (2020a) UEFA Calls meeting of European football stakeholders. UEFA, 12 March. Available at: https://www.uefa.com/insideuefa/about-uefa/news/025b-0f8e769170fb-415a0753a4e8-1000--uefa-calls-meeting-of-european-football-stakeholders/(accessed 27 May 2020).

[bibr52-10126902211020169] UEFA (2020b) UEFA Postpones EURO 2020 by 12 months. UEFA, 17 March. Available at: https://www.uefa.com/insideuefa/about-uefa/news/newsid=2641071.html (accessed 27 May 2020).

[bibr53-10126902211020169] Wardman JK Lofstedt R (2020) COVID-19: Confronting a new world risk. Journal of Risk Research 23(7–8): 833–837.

[bibr54-10126902211020169] Weed M (2020) The role of the interface of sport and tourism in the response to the COVID-19 pandemic. Journal of Sport & Tourism 24(2): 79–92.

[bibr55-10126902211020169] WHO (2020) Listings of WHO’s response to COVID-19. WHO. Available at: https://www.who.int/news/item/29-06-2020-covidtimeline (accessed 12 January 2021).

[bibr56-10126902211020169] Włoch R (2012) UEFA As a new agent of global governance: A case study of relations between UEFA and the polish government against the background of the UEFA EURO 2012. Journal of Sport and Social Issues 37(7): 297–311.

[bibr57-10126902211020169] Włoch R (2020) Two dynamics of globalization in the context of a sports mega-event: The case of UEFA EURO 2012 in Poland. Globalizations 17(1): 45–59.

[bibr58-10126902211020169] Zinn JO (2020) ‘A monstrous threat’: How a state of exception turns into a ‘new normal’. Journal of Risk Research 23(7–8): 1083–1091.

